# Adaption and psychometric evaluation of the Hindi version of Neck Disability Index in the rural population of Northern India: A cross cultural study

**DOI:** 10.12688/f1000research.142451.1

**Published:** 2023-12-18

**Authors:** Mohammad Sidiq, Arunachalam Ramachandran, Balamurugan Janakiraman, Faizan Zaffar Kashoo, Aksh Chahal, Janvhi Singh, Yousef Almotairi, Abdul Aziz Almotairi, Mohammad Miraj, Sai Jaya Prakash CH, Krishna Reddy Vajrala, Ramprasad Muthukrishnan, Praveen Kumar Kandakurti

**Affiliations:** 1 Department of Physiotherapy, School of Allied Health Sciences, Madhav University, Abu Road Sirohi Rajasthan, 307026, India; 2 Department of Physiotherapy, School of Allied Health Sciences, Galgotias University, Greater Noida, 203201, India; 3 SRM College of Physiotherapy, Faculty of Medicine and Health Sciences, SRM Institute of Science and Technology (Deemed to be University), Kattankulathur, Tamil Nadu, 603203, India; 4 Department of Physical Therapy and Health Rehabilitation, College of Applied Medical Sciences, Majmaah University, Al Majmaah, Riyadh Province, 11932, Saudi Arabia; 5 Department of Physiotherapy, NIMS University, Jaipur, Rajasthan, 303121, India; 6 Rehabilitation Medicine Department, Northern Area Armed Forces Hospital, Hafar Al Batin, Eastern Province, 31991, Saudi Arabia; 7 PDS Institute of Physiotherapy, Kaloji Narayana Rao University of Health Sciences, Purani Haveli, Hyderabad, 506007, India; 8 College of Health Sciences, Gulf Medical University, Ajman, Al Jurf, 4184, United Arab Emirates

**Keywords:** Translation, Psychometric testing, Hindi, Neck pain, Neck disability index, Rural India

## Abstract

**Background:**

To ensure the validity and therapeutic utility of the Neck disability index (NDI) scale, translations, cultural adaptations and psychometric evidence is necessary. This study aimed to address the absence of a suitable and validated Hindi version of the NDI for the rural population. The specific objectives were to translate, adapt, and evaluate the psychometric properties of the newly developed Hindi version of the NDI.

**Methods:**

Following guidelines provided by the American Association of Orthopedic Surgeons, the original English NDI scale was cross-culturally adapted into Hindi. The adaptation process included translations (forward and backward), expert committee review, pre-testing and cognitive debriefing with 30 individuals experiencing chronic non-specific neck pain. The outcome of this process was the creation of the Hindi version of the NDI, termed NDI-Hi. Subsequently, NDI-Hi was administered to 211 participants with neck pain from multiple centers for psychometric testing. The evaluation involved test-retest reliability over a 48-hour interval, factor analysis, assessment of internal reliability measures, and criterion-related validity by comparing it with the NPAD-Hindi version.

**Results:**

The NDI-Hi version exhibited favorable psychometric properties, including good test-retest reliability with an intra-class correlation coefficient (ICC) of 0.87. Internal consistency of the scale was high, indicated by Cronbach’s alpha coefficient (α) of 0.96. The standard error of measurement (SEM) was determined to be 2.58, and the minimal detectable change (MDC) was calculated to be 7.15. Furthermore, the NDI-Hi showed significant correlation with the NPAD-Hindi version, with a correlation coefficient (rho) of 0.86, and a p-value of less than 0.001.

**Conclusions:**

The NDI-Hi demonstrated validity and reliability as an outcome tool for assessing neck disability. It can be effectively utilized in clinical practice and research settings involving Hindi-speaking individuals with chronic non-specific neck pain. The adapted scale is particularly well-suited for the rural Northern Indian Hindi-speaking population.

## Introduction

Non-specific neck pain is characterized by lack of pathognomonic signs and symptoms ( Bernal-Utrera *et al.*, 2020) and neck pain is the second biggest contributor to the global disability-adjusted life years among musculoskeletal disorders ( Blyth *et al.*, 2019). Around 50% of adults experience neck pain annually, leading to a poorer quality of life ( Safiri *et al.*, 2017). Disability related to neck pain represent a substantial socioeconomic burden ( *e.g.*, health care utilization, work absenteeism, and lost productivity) ( Manchikanti *et al.*, 2009). In 2016, among the 154 conditions, low back and neck pain had the highest health-care spending in the United States estimated at USD 134.5 billion ( Dieleman *et al.*, 2020). In rural India, the average expenditure on healthcare per family in Indian rupees is INR 3,040.82 per month ( O. Article, 2018), but musculoskeletal disorders are yet to be included in National Health Policy 2017 ( Principles *et al.*, n.d.). Most people living with non-specific neck pain reported experience of physical ( *e.g.*, pain and disability) and psychological ( *e.g.*, anxiety, depression, and fear avoidance beliefs) concerns ( Simula *et al.*, 2019). Patient-reported outcome measures (PROMs) are commonly used in research and clinical settings to report physical and psychological problems in individuals with neck pain ( Wirth *et al.*, 2016) ( Lee *et al.*, 2015). The NDI is a reliable, valid, and commonly used PROM to evaluate physical problems (pain and disability) among individuals with neck pain, which has been translated into many different languages like; Turkish ( A. T. V. Study, 2008), Brazilian ( Cook *et al.*, 2006), Portuguese ( Cook *et al.*, 2006), German ( Awaji, 2016), French, Swedish (though modified) ( Lee *et al.*, 2006), Korean ( Song *et al.*, 2010), Iranian ( Mousavi *et al.*, 2007), Dutch ( Reneman, 2012), Greek ( Trouli *et al.*, 2008), Italian ( Monticone *et al.*, 2012), Arabic ( Khair *et al.*, 2020), Japanese ( Nakamaru *et al.*, 2012), Polish ( Misterska *et al.*, 2011), Thai, Punjabi ( Sandal *et al.*, 2021) and Marathi language ( A. M. V. Study, 2015). Translating a widely used questionnaire allows comparisons of different populations, clinical trials to determine the functional ability of the population with neck pain in India and also permits comparison or exchange of findings beyond cultural and language barriers. Questionnaires meant to be used across cultures must be translated linguistically to improve understanding and adapted to the cultural context to maintain the content validity of the tool. Out of the total population of 1.3 billion Indians, about 0.322 billion native and 0.270 billion non-native speakers speak Hindi as per the National Census of India 2011 ( Principles *et al.*, n.d.).

In our extensive search, authors found one published work that aimed to translate, adapt and perform test-retest reliability, validity of the Hindi-version of NDI ( Shakil *et al.*, 2011), but the authors reported of not conducting the validity test due to feasibility constraints. We found that the authors of the previous version did not follow steps recommended or guidelines for the process of cross cultural adaptation of self-reported measures ( Beaton *et al.*, 2000) and most of the psychometric properties analysis, dimensionality factor analysis, responsiveness (MDC, Minimal Detectable Change) were are neither performed nor mentioned, living behind a methodological gap that has led to considerably decreased utility of Hindi-version of NDI in research and clinical set up. Further, there is compelling evidence from a review that this published work lacks methodological quality in the process of translation, which implies conduction of further research to develop a reliable, validated, and tested Hindi version of NDI. In addition, the previous version also has no mention of permission from the original author of the NDI tool ( Pellicciari *et al.*, 2016). To improve the inclusivity in clinical set up and research, it is important that the translation process, adaptation understanding the cultural norms, values and behaviors are prioritized. Further, it is mandate to follow as per the steps for recommended guidelines for the process of cross-cultural adaptation of self-reported measures and rigorous psychometric properties analysis as recommended by Consensus-based Standards for the selection of health Measurement Instruments (COSMIN) ( Mokkink *et al.*, 2010).

As the occurrence of neck pain continues to rise, especially among native Hindi speakers, having a well-validated Hindi version of the NDI will enhance its clinical applicability and, most significantly, foster the inclusion of Hindi-speaking individuals suffering from neck pain in rural India but lacking proficiency in the English language. Therefore, this study intended to translate and adapt the original version of NDI into Hindi and assess its psychometric properties among the Hindi-speaking people with chronic non-specific neck pain (CNSNP). Furthermore, objectives include translating NDI into the Hindi language and testing the Hindi version of the NDI for its reliability and validity and responsiveness among patients with CNSNP.

## Methods

The present study is an observational cross-sectional study that involves two phases, the first phase includes the translation of the NDI and the second phase is where the psychometric properties were tested. The translation, adaptation, and psychometric testing of the Hindi version of NDI in this study was performed for the tool requirement of a future registered clinical trial among Hindi speaking rural North Indian population (Clinical Trials Registry of India, CTRI/2021/08/036040).

The study adhered to the SAGER guidelines for reporting sex and gender information. Sex and gender differences were not taken into consideration for the design of the study ( Heidari *et al.*, 2016).

### Ethical approval

This study was approved by the Institutional Review Board of the Madhav University, Rajasthan, (Ref. No: MU/IEC/21/14; dated 24/07/2021) and written informed consent for participation was obtained before patient recruitment. All procedures were conducted in accordance with the declaration of Helsinki. The purposes and the importance of the study were clarified to each participant. Participants were free to refuse to participate or answer any of the questions throughout the course of the study to ensure data confidentiality at all levels of the study, names of participants and any personal identifiers were not included. The permission to develop NDI-Hindi version was obtained from the author of the original version Professor Howard Vernon.

### Participants

This study was conducted at the Physiotherapy outpatient department (OPD) of Sanskriti University Mathura, India and Jeevan Jyoti Hospital Mathura in the Northern State of India (Mathura city, Uttar Pradesh) from August 2021 to June 2022. Hence, the study protocol was presented to the Ethical member at the Madhav University and approval obtained. Further, the Ethical approval letter and the study proposal was submitted to the Clinical Trial Registry of India (Government of India) CTRI, which verified the application and granted permission to conduct the definitive study. The study settings are the associate centers of Madhav University, and proper permission was obtained from the two centers to conduct the study. A total of 220 participants with CNSNP were recruited across two centers, of which 211 participants (114 females and 97 males) completed all participant-reported socio-demographic data, clinical information using face to face interviews as the participants in general visit the study center for consultations and physical rehabilitation, hence we decided to use interview method to improve the quality of data collected. by trained assessors. Neck Disability Index – Hindi version (NDI-Hi) and Neck Pain and Disability Scale (NPAD) ( Agarwal & Allison, 2006). This study followed recommendations of best practices for developing and validating scales ( R. Article, 2017), which suggests at least 5-10 participants per item in the tool as recommended by the COSMIN checklist ( Mokkink *et al.*, 2010; Sidiq, 2023). A sample size of 220 subjects was considered to improve the accuracy of the findings. Both, men and women, aged 18 years old and above living with CNSNP > 3 months duration were included in the study. Individuals with acute neck pain, diseases causing neck disability, previous cervical spine injury, prolapsed intervertebral disc disease, traumatic vertebral fractures, neck surgery, clinically recognizable cognitive impairment, pregnancy and those who could not read and speak Hind were excluded from the study.

### Instruments

*Neck Disability Index (NDI)*

The NDI, originally developed by Vernon and Mior ( Vernon & Mior, 1991) is a 10-item or section patient-reported outcome measure designed to assess neck pain and pain-related disability. The tool consist of 10 sections designed to self-report on how neck pain impacts the activities of daily life, the domains are; intensity of pain, personal care (dressing, washing), lifting heavy and light weights, reading, headaches, concentration, work, driving, sleeping, and recreation. Each section consists of six activity-based exclusive responses expressing progressive levels of functional disability. Item scores range from 0 (no disability) to 5 (total disability) and Vernon also established categorization of scores as; 0 to 4 no disability, 5 to 14 mild disability, 15 to 24 moderate disability, 25 to 34 severe disability, and ≥ 35 complete disability. The total score is out of 50 ( Vernon & Mior, 1991) and is also frequently normalized to 100 and reported as a percentage. The NDI (one-dimensional questionnaire) measures a single construct, and the questionnaire has demonstrated moderate differences in validity and reliability with a different patient population ( Pietrobon *et al.*, 2002).

The NDI© is protected by international copyright, with all rights reserved to Dr Howard Vernon. Do not use without permission. For information on, or permission to use the **NDI©**, please contact Mapi Research Trust at https://eprovide.mapi-trust.org. NDI © Dr Howard Vernon, 1991. All Rights Reserved. The users who do not receive specific funding for their use of the questionnaire can download the **NDI©** from ePROVIDE™, using the “online distribution” process.

*Neck Pain and Disability Scale (NPAD)*

The NPAD scale is a 20-item multi-dimensional questionnaire designed to assess neck pain and disability. The items are scored along a 10 cm visual analog scale (VAS) with the scores ranging from 0 to 5 in VAS. A higher score indicates greater disability. The Hindi version of NPAD has been cross-culturally translated and validated among 64 Indian individuals with cervical radiculopathy. The Hindi version of NPAD has been psychometrically tested and shown to have acceptable internal consistency and validity ( Agarwal & Allison, 2006).

### Translation and cross-cultural adaptation process

**Figure 1.**
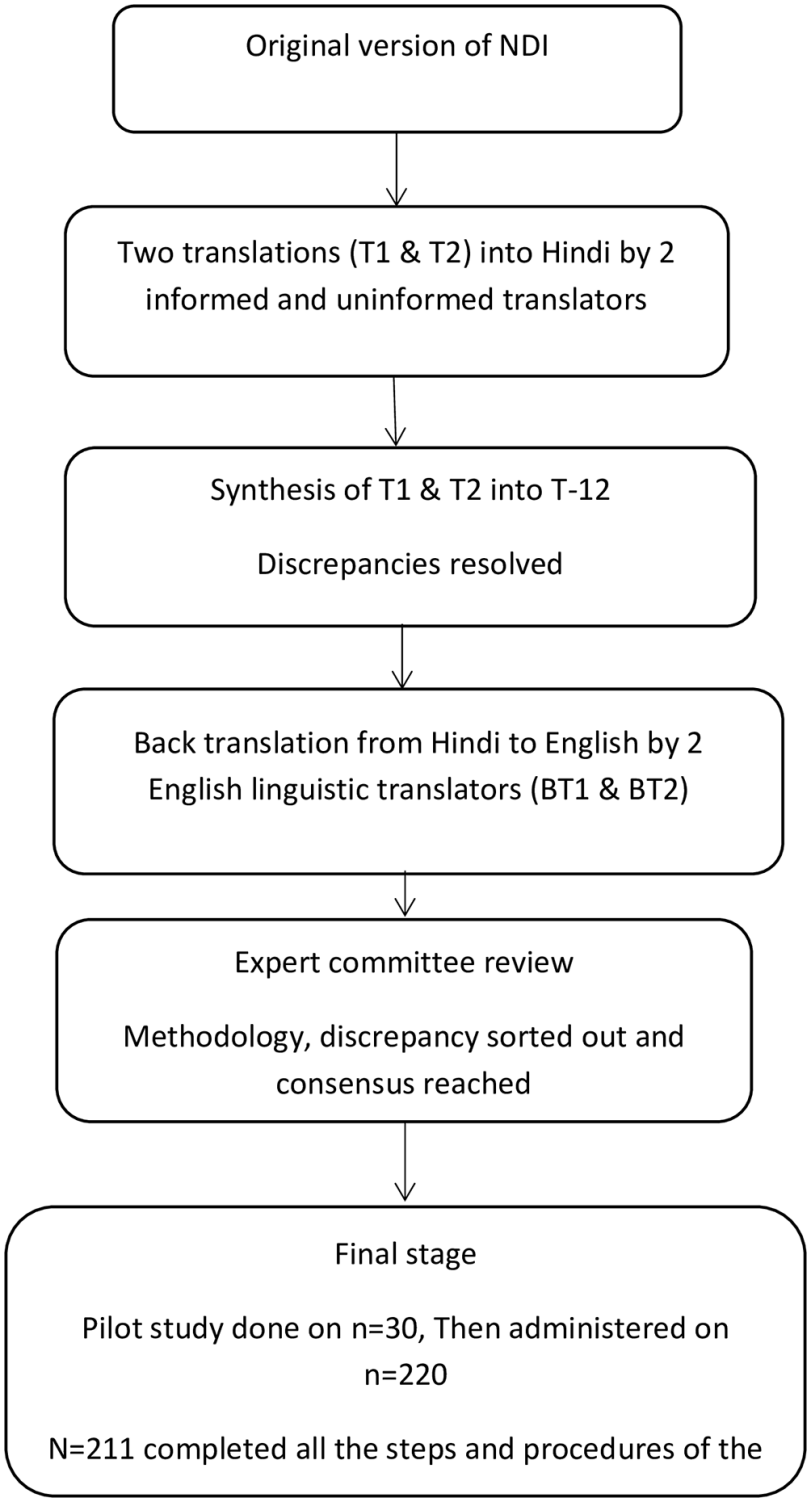
Study flowchart. NDI, Neck disability index.

Tool adaptation and translation was conducted as per the guidelines suggested by Beaton *et al.*, 2000 ( Beaton *et al.*, 2000). The translation and adaptation process for NDI consists of the following steps; forward translation, synthesis, backward translation, expert committee review, and synthesis, tool pretesting, and submission and appraisal of the written reports by the translation-adaptation coordinating committee ( Beaton *et al.*, 2000). In step one, forward translation was performed from English to Hindi language by two Hindi native translators (a professional translator and a post-graduate employee working in education department) who were fluent in the English language. One independent translator was blinded to process of forward translation of NDI. This was done to make sure the equivalency from a therapeutic point of view rather than literal equivalence. The other translator was informed about the purpose of the study and the concepts being studied. This was for contemplating the language used by the population and spotlight terms in the original questionnaire, the translation of which might have been obscure. Two forward-translated documents (T1 and T2) were produced. In step two, the independently translated T1 & T2 documents were shared among the two translators to synthesize NDI-Hi (version 1). Any inconsistencies, differences in the concepts and/or meaning, elusive wordings were sorted by discussion and agreement. In step three, the first draft NDI-Hi was back-translated to English language by two bilingual independent translators (native Hindi language speaking teachers with master’s degree in English) and not familiar with the construct being assessed and blinded to the process of forward translation. After discussion and agreement between back translators and the principal investigator (MS), a third translation was performed. In step four, all three documents were presented to the expert panel for review and discussion. The expert committee was comprised of four translators, two review committee experts from the university and two senior physiotherapy academicians who reviewed the Hindi version of the questionnaire. The panel discussed clarity, relevance, modifications, comprehension and synthesized the pre-final version for field testing Figure 1.

### Pilot study and modifications

In step five, upon approval of the panel, NDI-Hi was piloted on 30 individuals with CNSNP (19 females and 11 males). A cognitive (qualitative) debriefing was conducted to evaluate the understanding, clarity, language, cultural appropriateness, and acceptability of the NDI-Hi. As a result, the majority of participants reported a good understanding of the tool. However, 16 participants reported section eight ‘driving’ not applicable to them. Based on cognitive debriefing comments made by the participants, the committee agreed to reword section eight (driving) to fit the cultural context in India where most people do not drive car, rather travel by public and/or private transport and motorbike. For the participants who did not answer/drive, the total score was adjusted from 45 instead of 50 (multiplying 50/45). The final Hindi version of the NDI was then compared with the original one to achieve representational, fact-finding and contextual equivalence.

### Psychometric testing of the NDI-Hi

Psychometric properties were assessed using the final version of the NDI-Hindi ( Sidiq, 2023) on 211 patients with CNSNP for > 3 months duration across three centers. The test-retest reliability of the NDI-Hi version was determined by calculating Cronbach’s alpha with an alpha value of > 0.7 (deemed acceptable), > 0.8 (considered good), and > 0.9 (considered excellent) to grade the internal consistency ( Taber, 2018). The minimum sample size required for two-tailed Cronbach’s alpha test was calculated using the Bonet formula based on assumptions; item in the scale (k) 10, power 0.90 (1 - β), type I error (α) 0.05, value of Cronbach’s alpha at null hypothesis (CA0) and expected value of Cronbach’s alpha (CA1) were identified at 0.0 and 0.8, respectively ( Bonett, 2002). The required sample size was 70. NDI-Hi was re-administered on about 50% of the participants (n = 110) at the interval of 48 hours to assess its test-retest reliability. This is more than the power calculated sample.

**Figure 2.**
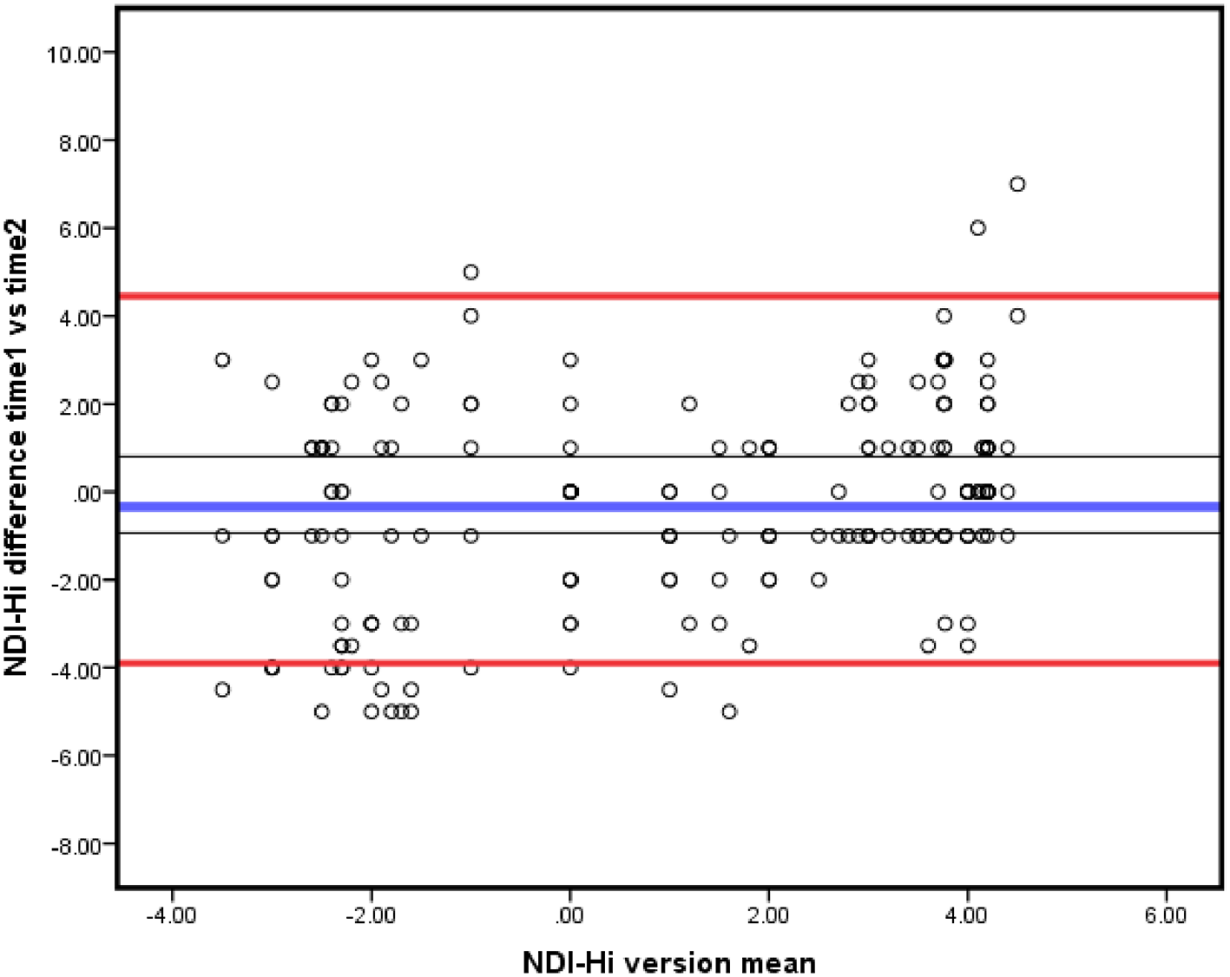
Bland-Altman plot of agreement between test and re-test scores of NDI-Hi. Bold blue line representing the mean of difference, the red lines representing 95% LOA and the grey lines representing the 95% CI of the mean of the difference. NDI-Hi, Hindi-version of the Neck disability index; LOA, limits of agreement.

To determine the dimensionality of the NDI-Hi, a stepwise exploratory factor analysis (EFA) was performed with Kaiser Meyer Olkin and Bartlett’s criteria with a retention rule of an Eigenvalue > 1 (items with loadings ≥ 0.4 were considered satisfactory). Independent factors were obtained with maximum likelihood using the Varimax rotation model. The floor and ceiling effects, distribution and completeness of items responses and missing values were examined for content validity. The concurrent validity method was used for the criterion-related validity; Pearson’s correlation analysis was done to establish relation between NDI-Hi and Visual Analogue Scale (VAS). The convergent construct validity was examined by comparing the NDI-Hi and Hindi version of NPAD using Spearman rank correlation coefficient (r). The r value of > 0.80 were considered excellent, and 0.61 to 0.80 very good. It is expected that the Hindi versions of NDI and NPAD would have moderate correlations since both tools measures the impact of neck pain on the functional status of the patients ( Agarwal *et al.*, 2006). Standard error of measurement (SEM) was calculated from the square root (SEM = SD √ (1-R)) of the within-subject variance from ANOVA for random-effect to evaluate the measurement error. MDC _95%_ was derived using the SEM to allow expression of the smallest magnitude of change that reveals the true change rather than measurement error by using the formula MDC _95%_ = 1.96 √2x SEM or 2.7*SEM. The Bland-Altman plot was also used to determine agreement ( Bland & Giavarina, 2015) ( Figure 2).

### Statistical analysis

IBM SPSS Statistics (RRID: SCR_016479) version 20 was used to analyze the data (SPSS 20, IBM, Armonk, NY, USA). Characteristics and observations of the quantitative and qualitative variables were summed using the mean, standard deviation (SD) and frequencies or relative frequencies. Normality of scores from different instruments was examined using one-sample Kolmogorov-Smirnov test. Internal consistency, test-retest reliability, and convergent validity of the two scales used in the analysis were examined. Authors in the present study were able to determine the internal consistency of the NDI-Hindi using Cronbach’s alpha index with values between 0.70 and 0.90 considered satisfactory. For test-retest reliability, interclass correlation coefficients (ICCs) and 95 percent confidence intervals (CIs) were determined. ICCs below 0.40 were considered low, those in the range of 0.4-0.70 were considered moderate, 0.70-0.90 were considered significant and values above 0.9 were considered exceptional respectively. The degree of correlation between NDI-Hindi and the NPAD at baseline was determined using Spearman’s rho correlation. The significance level was set at P < 0.05. According to the sex and Gender Equity in research (SAGER) guidelines, data analysis of sex/gender differences and/or similarities were performed.

## Results

### Participants

Demographic variables of the participants have been tabulated in Table 1 ( Sidiq *et al.*, 2023). All participants were assessed for their body mass index, duration of neck pain, pain medication, associated medical condition, marital status, employment status, level of education, income and mean and SD scores of NDI and NPAD. The majority of participants were female (n = 149, 70.6%), married (n = 152, 72.0%) and unemployed (n = 120, 56.9%).

**Table 1.** Socio-demographic and clinical characteristics of participants with CNNP for psychometric testing (n = 211).

Variables	n (%)	Mean (SD)
Age (years)		40.6 ± 10.5
Sex		
Male	62 (29.4)	
Female	149 (70.6)	
Education		
No formal schooling	18 (8.5)	
Primary	20 (9.5)	
Secondary	49 (23.2)	
Diploma/Skill trainings	34 (16.1)	
University level	90 (42.7)	
Duration of pain (months)		17.7 ± 18.1
< 6 months	42 (19.9)	
6 to 12 months	93 (44.1)	
>1 year	76 (36.0)	
1 ^st^ assessment		
NDI-Hi (0-50)		38.5 ± 5.6
VAS (0-10)		
2 ^nd^ assessment **(n = 110)**		
NDI-Hi (0-50)		39.1 ± 5.2
VAS (0-10)		
Mean difference test-retest **(n = 110)**		
NDI-Hi (0-50)		0.6 ± 3.8
VAS (0-10)		

CNNP, chronic non-specific neck pain; NDI-Hi, Hindi-version of the Neck disability index; VAS, visual analog scale.

### Participant’s response to NDI-Hi

Table 2 displays the mean scores extracted from the participants in 10 sections of the NDI-Hindi. Participants were moderately affected due to neck disorder and the mean rating on NDI was 32.02 (6.9) out of 50 in total. Principle component analysis revealed two-factor structures (physical dysfunction and neurological dysfunction) of NDI-Hindi. The physical dysfunction included pain intensity personal care, lifting, work, driving, reading and recreation were moderately to severely affected with a mean score ranging from 3.36-3.92, while neurological dysfunction that included headaches, concentration and sleeping were mildly affected with mean scores 2.3 and standard deviation of 0.6.

**Table 2.** Reliability (n = 211) of the NDI-Hi, internal consistency, test-retest reliability, and SEM (n = 110).

NDI domains (items)	T1 NDI-Hi mean ± SD	T2 NDI-Hi mean ± SD	ICC (95% CI) (n = 110)	SEM	MDC _95%_
Pain Intensity *	4.1 ± 1.0	4.2 ± 0.9	0.84	0.36	1.00
Personal Care **	3.8 ± 0.4	3.8 ± 0.4	0.885	0.13	0.35
Lifting **	3.9 ± 0.9	4.0 ± 1.0	0.917	0.29	0.81
Reading **	3.9 ± 0.9	4.0 ± 0.6	0.896	0.19	0.52
Headaches *	3.7 ± 0.6	3.8 ± 0.9	0.845	0.35	0.98
Concentration *	3.8 ± 0.9	3.8 ± 0.3	0.845	0.15	0.41
Work *	3.7 ± 0.5	3.8 ± 0.8	0.845	0.35	0.98
Driving *	3.8 ± 0.9	3.9 ± 0.7	0.869	0.25	0.69
Sleeping **	3.9 ± 0.9	4.0 ± 1.0	0.881	0.34	0.96
Recreation **	3.6 ± 0.4	3.7 ± 0.5	0.873	0.17	0.47
**Total Score (50)**	**38.5**	**39.1**	**0.87**	**2.58**	**7.15**

NDI-Hi, Hindi-version of the Neck disability index; SEM, Standard Error of Measurement; ICC, intra-class correlation coefficient; MDC, minimal detectable change; SD, standard deviation; T1, Test one of NDI-HI; T2, re-test of NDI-Hi; CI, Confidence Interval.

* Indicates <0.05.

** Indicates <0.01.

### Internal consistency of the NDI-Hi

The Cronbach’s alpha reliability was 0.962, which indicated that respondents were able to read and comprehend the 10 NDI-Hindi items well. However, all 10 indicators demonstrated a meaningful adjusted total association with their total scores. When these indicators were removed from the total, all of the items exhibited a corrected item-total correlation greater than 0.75, showing reasonably substantial shared covariance ( Table 2).

### Test-retest reliability of NDI Hi

Test-retest reliability was assessed seven days after the initial assessment and was found to be excellent. The test-retest reliability was excellent for the overall NDI-Hindi and average Interclass correlation coefficient (ICC) was 0.893, with a 95% Confidence interval (CI) (0.795, 0.937) (p < 0.001) ( Table 2). The mean of item variances was 0.648, with inter-item covariance’s mean of 0.465 ( Table 3).

**Table 3.** Internal consistency of the NDI-Hi if item deleted (n = 211).

Item - Total Statistics				
Items in NDI	Scale Mean if Item Deleted	Scale Variance if Item Deleted	Corrected Item-Total Correlation	Cronbach’s Alpha if Item Deleted
**Pain Intensity**	28.6	39.01	0.80	0.95
**Personal Care**	28.6	40.7	0.85	0.95
**Lifting**	28.4	37.1	0.92	0.95
**Work**	28.6	40.3	0.93	0.95
**Headaches**	29.6	40.8	0.82	0.95
**Concentration**	29.6	40.8	0.82	0.95
**Sleeping**	29.6	40.8	0.82	0.95
**Driving**	28.3	37.7	0.86	0.95
**Reading**	28.3	36.1	0.85	0.95
**Recreation**	28.1	39.1	0.75	0.96

NDI-Hi, Hindi-version of the Neck disability index.

The overall Cronbach’s alpha of 10 items was 0.962.

### Construct validity of NDI-Hi

**Figure 3.**
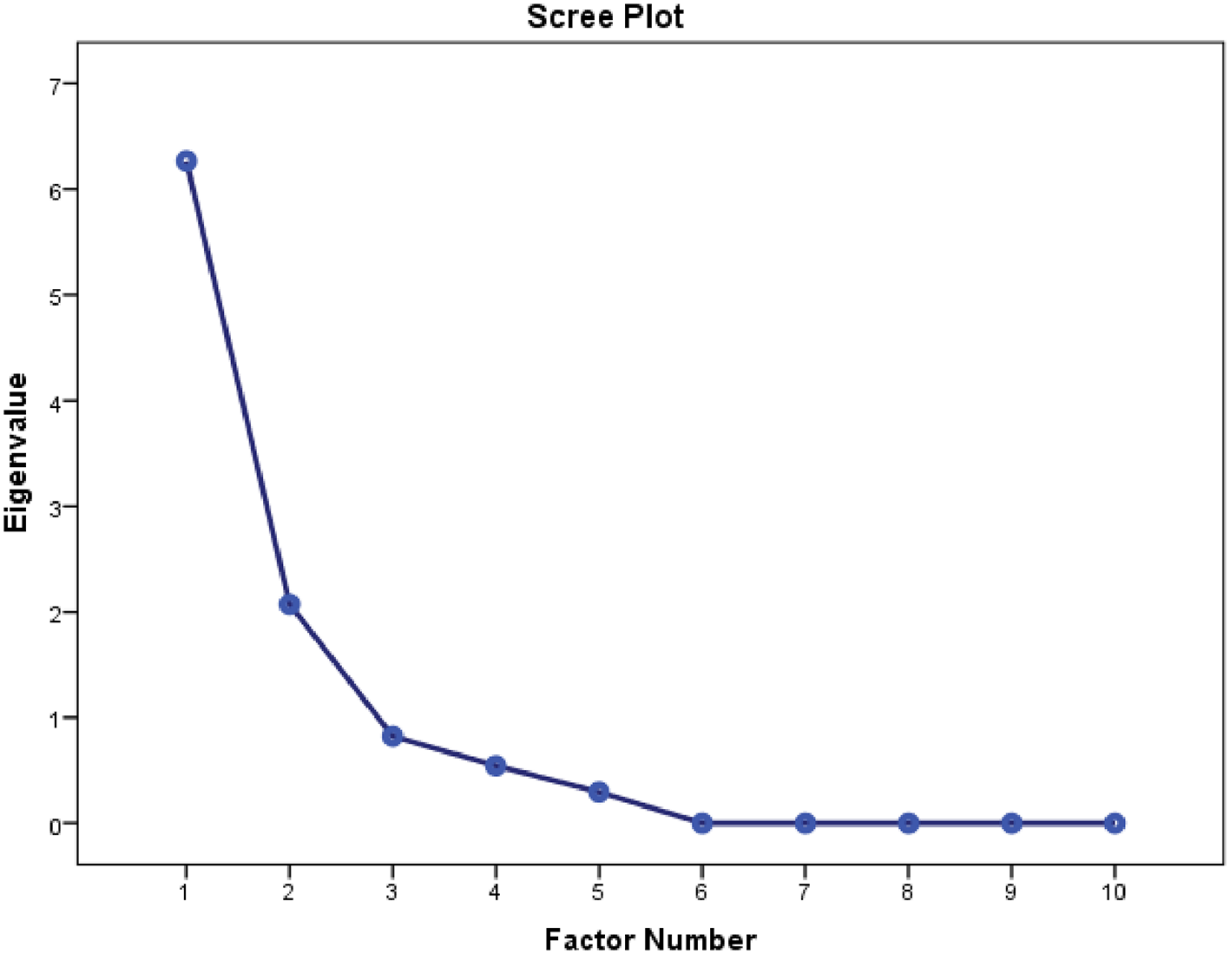
Scree plot indicating factor loading for NDI-Hi. NDI-Hi, Hindi-version of the Neck disability index.

Construct validity of the NDI-Hindi was estimated using principal component analysis (factor analysis) with varimax rotation ( Table 4). Data met the assumptions (Kaiser–Meyer Olkin = 0.826) of sample adequacy and Bartlett’s test of sphericity. The total variance explained by two factors was 87.5%. The analysis found a two factor structure of scale; Factor 1 included the first seven items of the questionnaire (77.3% of variance), Factor 2 included three items (10.2% of variance) ( Table 5). Factor loading was determined using a scree plot ( Figure 3).

**Table 4.** Construct validity of NDI-Hi.

NDI Items	Pain Intensity	Personal Care	Lifting	Work	Headaches	Concentration	Sleeping	Driving	Reading
Pain Intensity									
Personal Care	0.760 **								
Lifting	0.898 **	0.917 **							
Work	0.771 **	0.931 **	0.922 **						
Headaches	0.655 **	0.722 **	0.742 **	0.783 **					
Concentration	0.655 **	0.722 **	0.742 **	0.783 **	1.000 **				
Sleeping	0.655 **	0.722 **	0.742 **	0.783 **	1.000 **	1.000 **			
Driving	0.857 **	0.843 **	0.934 **	0.839 **	0.663 **	0.663 **	0.663 **		
Reading	0.703 **	0.880 **	0.906 **	0.957 **	0.764 **	0.764 **	0.764 **	0.833 **	
Recreation	0.768 **	0.771 **	0.777 **	0.814 **	0.695 **	0.695 **	0.695 **	0.655 **	0.772 **

NDI-Hi, Hindi-version of the Neck disability index.

** Correlation is significant at the 0.01 level (2-tailed).

* Correlation is significant at the 0.05 level (2-tailed).

**Table 5.** Principle factor analysis for two components on the Varimax rotation for the NDI-Hi.

Items of NDI	Component	
	Physical Dysfunction	Neurological Dysfunction
**Lifting**	0.910	
**Driving**	0.904	
**Pain Intensity**	0.850	
**Personal Care**	0.811	
**Work**	0.788	
**Reading**	0.735	
**Concentration**		0.921
**Sleeping**		0.921
**Headaches**		0.921

NDI-Hi, Hindi-version of the Neck disability index.

Extraction Method: Principal Component Analysis.

Rotation Method: Varimax with Kaiser Normalization (KMO).

### Convergent validity of NDI-Hi

The scores of NDI-Hindi showed excellent correlation with the total scores on Hindi-validated version of NPAD scale. Scores on neck pain and disability scale showed a significant positive correlation with all 10 items of NDI, with the highest correlation with lifting item (0.917, p = 001) and a least but significant correlation with the concentration item and sleeping (0.845, p = 0.001) ( Table 6).

**Table 6.** Spearman's rho correlations of the NDI-Hi with the sub scales of NPAD.

NDI items	Neck Pain and Disability Scale
**Pain Intensity**	0.840 **
**Personal Care**	0.885 **
**Lifting**	0.917 **
**Work**	0.896 **
**Headaches**	0.845 **
**Concentration**	0.845 **
**Sleeping**	0.845 **
**Driving**	0.869 **
**Reading**	0.881 **
**Recreation**	0.873 **

NDI-Hi, Hindi-version of the Neck disability index; NPAD, Neck Pain and Disability Scale.

** Correlation is significant at the 0.01 level (2-tailed).

* Correlation is significant at the 0.05 level (2-tailed).

## Discussion

This study aimed to translate and adapt the original English version of NDI into the national language of India (Hindi) and test the psychometric properties of the NDI-Hi version. The recommended guidelines were followed during translation and adaptations ( Beaton *et al.*, 2000). The translation, adaptation, and synthesis of NDI-Hi was easy and accepted by the Hindi speaking people with CNSNP. Overall findings from this study indicate NDI-Hi to be a reliable and valid patient-reported outcome measure that could be used by researchers and clinicians to assess disability and functional limitations caused by neck pain among the Hindi-speaking population who are unable to read or write in the English language.

NDI-Hi demonstrated excellent internal consistency and Cronbach α of 0.89, which is comparable to the reports of previous works (0.74–0.97) and explains sample adequacy ( Wu *et al.*, 2010). In this study, the time interval for the test-retest re-administration was set at 48 hours, a duration that is likely to result in minimal alterations in the patient’s clinical condition and minimal memory-related effects. Likewise, NDI-Hi also showed good internal consistency, indicating good reliability. The acceptability of the NDI-Hi showed that all the items were below the threshold recommended for floor effects (20%). However, the ceiling effect of the pain intensity was found to be marginally (21.6%) higher than the threshold recommended. Similarly, previous studies reported no floor or ceiling effects with the only exception being the intensity of pain ( Nakamaru *et al.*, 2012; Shashua *et al.*, 2016; Trouli *et al.*, 2008; Farooq *et al.*, 2017). Though this phenomenon is not unique, a study on the Malay version of NDI reported moderate responsiveness of pain intensity and also explains the complexity in quantifying pain ( Lim *et al.*, 2020).

In our study, the pilot test conducted to check the feasibility and acceptability of the translated NDI-Hi version found that the majority of participants were not responding to the item on driving and the same was discussed in the cognitive debriefing. In India, the majority of individuals travel using motorbikes, public transportation, and private vehicles, with a relatively small portion choosing to commute by car. Moreover, when section eight, which pertains to driving, is culturally adapted to encompass driving, riding, or traveling, it significantly enhances the responses to this section, reducing the occurrence of missing values related to driving in various studies ( Cook *et al.*, 2006; Lee *et al.*, 2006). SEM (< 0.4) and MDC _95%_ of the NDI-Hi version indicated an absolute reliability and genuine agreement of repeated measurements, and suitability of NDI-Hi for clinical and research uses. MDC ^95%^ of 7.15 (score range 0-50) will guide clinicians and researchers of true change in NDI-Hi scores. Scores at or above MDC value indicate meaningful clinical change in patients. MDC value of NDI-Hi is comparable to the values reported by other researchers and smaller (precise) than other studies ( Pool *et al.*, 2007; Young *et al.*, 2009; Trouli *et al.*, 2008; Farooq *et al.*, 2017; Maki *et al.*, 2014; Shashua *et al.*, 2016). The smallest detectable change (SDC) and minimally important difference (MIC) reported by the Dutch version was 11.5, and 31.7 respectively, the Italian version NDI showed MIC of 10, and a methodological review conducted including 19 NDI versions reported only these three versions of NDI mentioned MIC ( Yao *et al.*, 2019). The MDC reported in this study is 7.15, which is relatively lower than MDC reported elsewhere, and this is suggestive that even a smaller change in the Hindi version of NDI shall be considered as an important yardstick of clinical relevant hence, offers a sensitive outcome tool even in case of less powered sample or individual level studies.

The validity of NDI-Hi was conducted by qualitative cognitive debriefing to establish content validity and correlation test of the scale with the NPAD Hindi version to evaluate the construct convergent validity ( Brod *et al.*, 2009). Based on the pilot study and cognitive briefing, rewording of item eight was done, which improved the understanding, acceptance and response rate. Factor analyses resulted in a hypothesized 2-factor solution for NDI-Hi represented as physical dysfunctions and neurological dysfunctions. These factors explained 69.51% of the total variance, and the variance reported was comparable to some previous studies ( Nakamaru *et al.*, 2012). The Hindi versions of NDI and NDAP appear to measure a similar construct. The NDI is a condition-specific patient-reported outcome measure (PROM) and it is frequently used in researches exploring intervention efficacy among individuals with neck pain. Similarly, psychometric properties of NDI-Hi propose it as a suitable PROM for Hindi-speaking individuals with neck pain. Nonetheless, suitability of NDI in patients with whiplash injury, social and emotional is questioned by Hoving *et al.*, highlighting limitation of the NDI that lacks elements to address these problems ( Hoving *et al.*, 2003).

### Study limitations

This study recruited samples beyond the recommended guidelines for cross-cultural adaptations of outcome measures and the required power calculated sample for test-retest reliability, which improved the statistical power of the psychometric evaluation of the NDI-HI version. However, there are few limitations to mention; this study is a cross-sectional design and hence any correlations should not be interpreted as causal effects. This study used only a self-reported questionnaire, the relationship between the activity limitations of neck pain (NDI) and physical tests were not considered and the study was done among rural northern Indian population only.

## Conclusions

The English version of NDI was successfully adapted to suit the rural Indian cultural context and translated to the Hindi-India version. The Hindi version of the NDI-Hi tool has healthy psychometric properties and appears to be suitable for use in epidemiological studies and clinical trials among Hindi language speakers.

## Data Availability

Figshare: NDI DATA SET. https://doi.org/10.6084/m9.figshare.24354952 ( Sidiq *et al.*, 2023). Figshare: COSMIN checklist for ‘Adaption and psychometric evaluation of the Hindi version of Neck Disability Index in the rural population of Northern India: a cross cultural study’. https://doi.org/10.6084/m9.figshare.24126609 ( Sidiq, 2023). Data are available under the terms of the Creative Commons Attribution 4.0 International license (CC-BY 4.0).

## References

[ref1] Agarwal S Allison GT : Reliability and validity of the Hindi version of the Neck Pain and Disability Scale in cervical radiculopathy patients. 2006. June 2014. 10.1080/09638280600641467 17071572

[ref2] Agarwal S Allison GT Agarwal A : Reliability and validity of the Hindi version of the Neck Pain and Disability Scale in cervical radiculopathy patients. *Disabil. Rehabil.* 2006;28(22):1405–1411. 10.1080/09638280600641467 17071572

[ref3] Article O : The Prevalence and Health Impact of Musculoskeletal Disorders among Farmers. 2018;485–491. 10.4103/mjdrdypu.mjdrdypu

[ref4] Article R : *Guidelines for developing, translating, and validating a questionnaire in perioperative and pain medicine.* SJA;2017. 10.4103/sja PMC5463570 28616007

[ref5] Awaji M : Epidemiology of Low Back Pain in Saudi Arabia. *J. Adv. Med. Pharm. Sci.* 2016;6(4):1–9. 10.9734/jamps/2016/24173

[ref6] Beaton DE Bombardier C Guillemin F : Guidelines for the Process of Cross-Cultural Adaptation of Self-Report Measures. *Spine.* December 15, 2000;25(24):3186–3191. 10.1097/00007632-200012150-00014 11124735

[ref7] Bernal-Utrera C Gonzalez-Gerez JJ Anarte-Lazo E : Manual therapy versus therapeutic exercise in non-specific chronic neck pain: A randomized controlled trial. *Trials.* 2020;21(1):1–10. 10.1186/s13063-020-04610-w 32723399 PMC7385865

[ref8] Bland U Giavarina D : Lessons in biostatistics. 2015;25(2):141–151. 10.11613/BM.2015.015 PMC4470095 26110027

[ref9] Blyth FM Briggs AM Schneider CH : The Global Burden of Musculoskeletal Pain — Where to From Here? *AJPH.* 2019;109(1):35–40. 10.2105/AJPH.2018.304747 PMC6301413 30495997

[ref10] Bonett DG : Sample Size Requirements for Testing and Estimating Coefficient Alpha. *J. Educ. Behav. Stat.* 2002;27(4):335–340. 10.3102/10769986027004335

[ref11] Brod M Tesler LE Christensen TL : Qualitative research and content validity: Developing best practices based on science and experience. *Qual. Life Res.* 2009;18(9):1263–1278. 10.1007/s11136-009-9540-9 19784865

[ref12] Cook C Richardson JK Braga L : Cross-cultural adaptation and validation of the Brazilian Portuguese version of the Neck Disability Index and Neck Pain and Disability Scale. *Spine.* 2006;31(14):1621–1627. 10.1097/01.brs.0000221989.53069.16 16778699

[ref13] Dieleman JL Cao J Chapin A : US Health Care Spending by Payer and Health Condition, 1996-2016. *JAMA.* 2020;323(9):863–884. 10.1001/jama.2020.0734 32125402 PMC7054840

[ref14] Farooq MN Mohseni-Bandpei MA Gilani SA : Urdu version of the neck disability index: A reliability and validity study. *BMC Musculoskelet. Disord.* 2017;18(1):1–11. 10.1186/s12891-017-1469-5 28388888 PMC5385030

[ref15] Heidari S Babor TF De Castro P : Sex and Gender Equity in Research: rationale for the SAGER guidelines and recommended use. *Res. Integr. Peer Rev.* 2016;1(1):1–9. 10.1186/s41073-016-0007-6 29451543 PMC5793986

[ref17] Hoving JL O’Leary EF Niere KR : Validity of the neck disability index, Northwick Park neck pain questionnaire, and problem elicitation technique for measuring disability associated with whiplash-associated disorders. *Pain.* 2003;102(3):273–281. 10.1016/S0304-3959(02)00406-2 12670669

[ref18] Khair U Shahid W Arshad N : Prevalence of nonspecific neck pain in dental practitioners of Lahore: A cross Prevalence of nonspecific neck pain in dental practitioners of Lahore: A cross sectional study. 2020 May.

[ref19] Lee H Nicholson LL Adams RD : Development and psychometric testing of Korean language versions of 4 neck pain and disability questionnaires. *Spine.* 2006;31(16):1841–1845. 10.1097/01.brs.0000227268.35035.a5 16845361

[ref20] Lee H Hübscher M Moseley GL : How does pain lead to disability? A systematic review and meta-analysis of mediation studies in people with back and neck pain. *Pain.* 2015;156:988–997. 10.1097/j.pain.0000000000000146 25760473

[ref21] Lim HHR Tan ST Tang ZY : Cross-cultural adaptation and psychometric evaluation of the Malay version of the Neck Disability Index. *Disabil. Rehabil.* 2020;1–7. 10.1080/09638288.2020.1758225 32374189

[ref22] Maki D Rajab E Watson PJ : Cross-cultural translation, adaptation, and psychometric testing of the Roland-Morris Disability Questionnaire into modern standard Arabic. *Spine.* 2014;39(25):E1537–E1544. 10.1097/BRS.0000000000000632 25271496

[ref23] Manchikanti L Singh V Datta S : Comprehensive review of epidemiology, scope, and impact of spinal pain. *Pain Physician.* 2009;12(4):E35–E70. 10.36076/ppj.2009/12/e35 19668291

[ref24] Misterska E Jankowski R Glowacki M : Cross-cultural adaptation of the Neck Disability Index and Copenhagen Neck Functional Disability Scale for patients with neck pain due to degenerative and discopathic disorders. Psychometric properties of the Polish versions. *BMC Musculoskelet. Disord.* 2011;12(1):84. 10.1186/1471-2474-12-84 21529360 PMC3108936

[ref25] Mokkink LB Terwee CB Knol DL : The COSMIN checklist for evaluating the methodological quality of studies on measurement properties: A clarification of its content. 2010. 10.1186/1471-2288-10-22 PMC2848183 20298572

[ref26] Monticone M Ferrante S Vernon H : Development of the Italian Version of the Neck Disability Index. 2012;37(17). 10.1097/BRS.0b013e3182579795 22487712

[ref27] Mousavi SJ Parnianpour M Montazeri A : Translation and Validation Study of the Iranian Versions of the Neck Disability Index and the Neck Pain and Disability Scale. *Spine.* 2007;32(26):825–831. 10.1097/BRS.0b013e31815ce6dd 18091478

[ref28] Nakamaru K Vernon H Aizawa J : Crosscultural adaptation, reliability, and validity of the Japanese version of the Neck Disability Index. *Spine.* 2012;37(21):E1343–E1347. 10.1097/BRS.0b013e318267f7f5 22789978

[ref29] Pellicciari L Bonetti F Di Foggia D : Patient-reported outcome measures for non-specific neck pain validated in the Italian-language: a systematic review. *Arch. Physiother.* 2016;6(1):9. 10.1186/s40945-016-0024-2 29340191 PMC5759912

[ref30] Pietrobon R Coeytaux RR Carey TS : Standard scales for measurement of functional outcome for cervical pain or dysfunction: A systematic review. *Spine.* 2002;27(5):515–522. 10.1097/00007632-200203010-00012 11880837

[ref31] Pool JJM Ostelo RWJG Hoving JL : Minimal clinically important change of the neck disability index and the numerical rating scale for patients with neck pain. *Spine.* 2007;32(26):3047–3051. 10.1097/BRS.0b013e31815cf75b 18091500

[ref32] Principles KP Goals SQ Thrust P : *NATIONAL HEALTH POLICY.* n.d.;2017.

[ref33] Reneman MF : Neck Pain and Disability Scale and Neck Disability Index: validity of Dutch language versions. 2012;93–100. 10.1007/s00586-011-1920-5 PMC3252449 21814745

[ref34] Safiri S Kolahi A Hoy D : Global, regional, and national burden of neck pain in the general population, 1990-2017: systematic analysis of the Global Burden of Disease Study 2017. *BMJ.* 2017;368. 10.1136/bmj.m791 32217608 PMC7249252

[ref35] Sandal D Jindal R Gupta S : Reliability and Validity of Cross Culturally Adapted Punjabi Version of NDI (NDI-P) in Patients with Neck Pain: A Psychometric Analysis. *Indian J. Orthop.* 2021;55(4):918–924. 10.1007/s43465-020-00280-7 34194648 PMC8192611

[ref36] Shakil H Khan SA Thakur PC : Test retest reliability and validity of Hindi version of Neck Disability Index in patients with neck pain. *Indian J. Physiother. Occup. Ther.* 2011;5:167–169.

[ref37] Shashua A Geva Y Levran I : Translation, validation, and crosscultural adaptation of the Hebrew version of the neck disability index. *Spine.* 2016;41(12):1036–1040. 10.1097/BRS.0000000000001445 27285662

[ref38] Sidiq M Janakiraman B Chahal A : NDI DATA SET.[Dataset]. *figshare.* 2023. 10.6084/m9.figshare.24354952.v1

[ref39] Sidiq M : COSMIN checklist (COnsensus-based Standards for the selection of health Measurement INstruments).[Dataset]. *figshare.* 2023. 10.6084/m9.figshare.24126609.v1

[ref40] Simula AS Jenkins HJ Holopainen R : Transcultural adaption and preliminary evaluation of “understanding low back pain” patient education booklet. *BMC Health Serv. Res.* 2019;19(1):1010–1011. 10.1186/s12913-019-4854-y 31888605 PMC6936060

[ref41] Song K Choi B Choi B : Cross-Cultural Adaptation and Validation of the Korean Version of the Neck Disability Index. *Spine.* 2010;35(20):1045–1049. 10.1097/BRS.0b013e3181df78e9 20436378

[ref42] Study, A. M. V: Cultural Adaptation, Reliability, and Validity of Neck Disability Index in Indian Rural Population. *Spine.* 2015;40(2). 10.1097/BRS.0000000000000681 25575090

[ref43] Study, A. T. V: The Cultural Adaptation, Reliability and Validity of Neck Disability Index in Patients With Neck Pain. *Spine.* 2008;33(11):362–365. 10.1097/BRS.0b013e31817144e1 18469684

[ref44] Taber KS : The Use of Cronbach’s Alpha When Developing and Reporting Research Instruments in Science Education. *Res. Sci. Educ.* 2018;48(6):1273–1296. 10.1007/s11165-016-9602-2

[ref45] Trouli MN Vernon HT Kakavelakis KN : Translation of the Neck Disability Index and validation of the Greek version in a sample of neck pain patients. *BMC Musculoskelet. Disord.* 2008;9:1–8. 10.1186/1471-2474-9-106 18647393 PMC2492862

[ref46] Vernon H Mior S : The Neck Disability Index: a study of reliability and validity. *J. Manip. Physiol. Ther.* 1991;14(7):409–415. 1834753

[ref47] Wirth B Humphreys BK Peterson C : Importance of psychological factors for the recovery from a first episode of acute non-specific neck pain - a longitudinal observational study. *Chiropr. Man. Therap.* 2016;24(1):9–10. 10.1186/s12998-016-0090-2 26985362 PMC4793758

[ref48] Wu S Ma C Mai M : Translation and validation study of Chinese versions of the neck disability index and the neck pain and disability scale. *Spine.* 2010;35(16):1575–1579. 10.1097/BRS.0b013e3181c6ea1b 20436379

[ref49] Yao M Xu B Tian Z : PT US CR. *Spine J.* 2019. 10.1016/j.spinee.2019.01.007

[ref50] Young BA Walker MJ Strunce JB : Responsiveness of the Neck Disability Index in patients with mechanical neck disorders. *Spine J.* 2009;9(10):802–808. 10.1016/j.spinee.2009.06.002 19632904

