# Supplementary material for: Adaption and psychometric evaluation of the Hindi version of Neck Disability Index in the rural population of Northern India: A cross cultural study
Source: F1000Res. 2024 Aug 8;12:1599. Originally published 2023 Dec 18. [Version 2] doi: 10.12688/f1000research.142451.2 (PMC11393535; doi:10.12688/f1000research.142451.2)
Supplement: Peer review history

# Reviewer report: Reviewer response for version 2

**DOI:** 10.5256/f1000research.169343.r312341

**Published:** 2024-09-12

**Authors:** Prem Venkatesan

**Affiliations:** 1 Manipal College of Health Professions, Bangalore, India

Authors addressed the queries raised and modified appropriately.

Is the work clearly and accurately presented and does it cite the current literature?

Partly

If applicable, is the statistical analysis and its interpretation appropriate?

Yes

Are all the source data underlying the results available to ensure full reproducibility?

Yes

Is the study design appropriate and is the work technically sound?

Partly

Are the conclusions drawn adequately supported by the results?

Yes

Are sufficient details of methods and analysis provided to allow replication by others?

Partly

Reviewer Expertise:

My area of expertise is musculoskeletal pain and cardiopulmonary rehabilitation

I confirm that I have read this submission and believe that I have an appropriate level of expertise to confirm that it is of an acceptable scientific standard.

# Reviewer report: Reviewer response for version 2

**DOI:** 10.5256/f1000research.169343.r312340

**Published:** 2024-08-23

**Authors:** Y. Ravi shankar Reddy

**Affiliations:** 1 Department of Medical Rehabilitation Sciences, College of Applied Medical Sciences, King Khalid University, Abha, Aseer Province, Saudi Arabia

The authors have thoroughly and successfully addressed the concerns raised, leading to a significant improvement in the manuscript's overall quality. Therefore, I am pleased to recommend that the manuscript be imdexed in its current form.

Is the work clearly and accurately presented and does it cite the current literature?

Partly

If applicable, is the statistical analysis and its interpretation appropriate?

Partly

Are all the source data underlying the results available to ensure full reproducibility?

Yes

Is the study design appropriate and is the work technically sound?

Yes

Are the conclusions drawn adequately supported by the results?

Partly

Are sufficient details of methods and analysis provided to allow replication by others?

Partly

Reviewer Expertise:

Rehabilitation, Physical Therapy

I confirm that I have read this submission and believe that I have an appropriate level of expertise to confirm that it is of an acceptable scientific standard.

# Response

**Published:** 2024-08-23

**Authors:** Dr Mohammad Sidiq

Thank you so much sir for your comments and approving the peer review. We express our sincere gratutude

# Reviewer report: Reviewer response for version 1

**DOI:** 10.5256/f1000research.156005.r291969

**Published:** 2024-07-11

**Authors:** Y. Ravi shankar Reddy

**Affiliations:** 1 Department of Medical Rehabilitation Sciences, College of Applied Medical Sciences, King Khalid University, Abha, Aseer Province, Saudi Arabia

Reviewer's Report

 Title:

 Adaption and Psychometric Evaluation of the Hindi Version of Neck Disability Index in the Rural Population of Northern India: A Cross-Cultural Study

  Abstract:

 The abstract provides a succinct overview of the study. However, it can be improved by adding more specific details to give readers a clearer understanding of the study's significance and outcomes. The background should specify the importance of validating the NDI for rural Hindi-speaking populations and how this fills a gap in current research. The methods section should detail the translation process more concisely, emphasizing the steps involved. The results section should highlight key statistics like the ICC, Cronbach's alpha, SEM, MDC, and correlation coefficients. Finally, the conclusions should reinforce the clinical applicability and importance of the NDI-Hi in rural settings.

  Introduction:

 The introduction effectively outlines the prevalence and impact of non-specific neck pain globally and in India, providing a solid rationale for the study. To enhance it further, include more recent statistics and studies to underscore the growing burden of neck pain. Highlight the unique challenges faced by rural populations in India, such as limited access to healthcare and language barriers. Additionally, the introduction should emphasize the importance of culturally adapting the NDI to ensure its relevance and accuracy for Hindi-speaking patients. This sets a clear context for the need for the NDI-Hi and its potential benefits.

  Methods:

 The methods section is comprehensive, detailing the translation and adaptation process meticulously. To improve clarity, consider breaking down the translation steps into sub-sections with clear headings for each stage: forward translation, synthesis, backward translation, expert committee review, and pilot testing. This would make the process easier to follow. The section on psychometric testing should provide more detail on the statistical methods used, such as the criteria for factor analysis and the rationale for the selected time intervals for test-retest reliability. Including a flowchart summarizing the entire process could also enhance clarity and readability.

  Results:

 The results section presents the findings effectively, with detailed statistical analysis. To make it more impactful, provide more context for the statistical figures, explaining what they mean in practical terms. For instance, elaborate on how the high Cronbach’s alpha and ICC values demonstrate the reliability and validity of the NDI-Hi. Include more descriptive statistics and graphical representations, such as bar charts or histograms, to visually summarize the data. Additionally, the demographic characteristics of the participants should be discussed in relation to the findings, highlighting any significant correlations or trends.

  Discussion:

 The discussion appropriately interprets the results, linking them back to the study's objectives and existing literature. To deepen the analysis, compare the psychometric properties of the NDI-Hi with those of other versions used in different cultural contexts. Discuss any unexpected findings or limitations encountered during the study, such as the challenges related to the driving item. Propose potential solutions or areas for future research to address these limitations. Furthermore, emphasize the clinical implications of the NDI-Hi, particularly how it can improve patient outcomes and research quality in rural Hindi-speaking populations.

  Conclusion:

 The conclusion succinctly summarizes the study's findings and their significance. To enhance it, reiterate the key strengths of the NDI-Hi, such as its high reliability and validity. Highlight the broader impact of having a validated Hindi version of the NDI, such as its potential to inform public health policies and improve clinical practices in rural India. Additionally, suggest specific applications of the NDI-Hi in both clinical and research settings, emphasizing its role in enhancing the understanding and management of neck pain among Hindi-speaking populations.

Is the work clearly and accurately presented and does it cite the current literature?

Partly

If applicable, is the statistical analysis and its interpretation appropriate?

Partly

Are all the source data underlying the results available to ensure full reproducibility?

Yes

Is the study design appropriate and is the work technically sound?

Yes

Are the conclusions drawn adequately supported by the results?

Partly

Are sufficient details of methods and analysis provided to allow replication by others?

Partly

Reviewer Expertise:

Rehabilitation, Physical Therapy

I confirm that I have read this submission and believe that I have an appropriate level of expertise to confirm that it is of an acceptable scientific standard, however I have significant reservations, as outlined above.

# Response

**Published:** 2024-07-17

**Authors:** Dr Mohammad Sidiq

Dear Editor,

 We express our sincere appreciation to the editor for the time and interest in this study.

 Dear reviewers,

**We appreciate and thank you for the valuable comments, suggestions, and interest in this research paper.**

 We have amended the corrections according to the comments and suggestions provided. In addition, Spelling, grammar, and sentence structure errors have been corrected and the manuscript is revised based on the journal style requirements.

*All the authors agreed on the changes made in response to the reviewer's comments.*

*Please find our responses below:*

 With regards,

 Dr Mohammad Sidiq

***Reviewer 2 comment***

 Abstract:

 The abstract provides a succinct overview of the study. However, it can be improved by adding more specific details to give readers a clearer understanding of the study's significance and outcomes.

*Authors’ response*

 The background section of the abstract is revised accordingly.

***Reviewer 2 comment***

 The background should specify the importance of validating the NDI for rural Hindi-speaking populations and how this fills a gap in current research.

 The methods section should detail the translation process more concisely, emphasizing the steps involved. The results section should highlight key statistics like the ICC, Cronbach's alpha, SEM, MDC, and correlation coefficients. Finally, the conclusions should reinforce the clinical applicability and importance of the NDI-Hi in rural settings.

*Authors’ response*

 Thank you, the changes are made according to the suggestions

***Reviewer 2 comment***

  Introduction:

 The introduction effectively outlines the prevalence and impact of non-specific neck pain globally and in India, providing a solid rationale for the study. To enhance it further, include more recent statistics and studies to underscore the growing burden of neck pain. Highlight the unique challenges faced by rural populations in India, such as limited access to healthcare and language barriers. Additionally, the introduction should emphasize the importance of culturally adapting the NDI to ensure its relevance and accuracy for Hindi-speaking patients. This sets a clear context for the need for the NDI-Hi and its potential benefits.

*Authors’ response*

 Thank you, amendments related to language barriers, and the need for a clear context related outcome measures is emphasized.

***Reviewer 2 comment***

 Methods:

 The methods section is comprehensive, detailing the translation and adaptation process meticulously. To improve clarity, consider breaking down the translation steps into sub-sections with clear headings for each stage: forward translation, synthesis, backward translation, expert committee review, and pilot testing.

 This would make the process easier to follow. The section on psychometric testing should provide more detail on the statistical methods used, such as the criteria for factor analysis and the rationale for the selected time intervals for test-retest reliability. Including a flowchart summarizing the entire process could also enhance clarity and readability.

*Authors’ response*

 Thank you, we have included a new figure 1 based on the suggestions- to clearly explain the steps into sub-sections.

 The rationale for using 48 hours interval for test retest reliability is now included with reference and a new flowchart added (figure 1).

 Details regarding factor analysis is now added under the statistical analysis section.

***Reviewer 2 comment***

 Results:

 The results section presents the findings effectively, with detailed statistical analysis. To make it more impactful, provide more context for the statistical figures, explaining what they mean in practical terms. For instance, elaborate on how the high Cronbach’s alpha and ICC values demonstrate the reliability and validity of the NDI-Hi. Include more descriptive statistics and graphical representations, such as bar charts or histograms, to visually summarize the data. Additionally, the demographic characteristics of the participants should be discussed in relation to the findings, highlighting any significant correlations or trends.  

*Authors’ response*

 We have added contents to explain the significance of findings.

***Reviewer 2 comment***

 Discussion:

 The discussion appropriately interprets the results, linking them back to the study's objectives and existing literature.

 To deepen the analysis, compare the psychometric properties of the NDI-Hi with those of other versions used in different cultural contexts. Discuss any unexpected findings or limitations encountered during the study, such as the challenges related to the driving item. Propose potential solutions or areas for future research to address these limitations. Furthermore, emphasize the clinical implications of the NDI-Hi, particularly how it can improve patient outcomes and research quality in rural Hindi-speaking populations.

*Authors’ response*

 We have now included statements related to the previous version of NDI and clinical  implication related contents in discussion section.

***Reviewer 2 comment***

 Conclusion:

 The conclusion succinctly summarizes the study's findings and their significance. To enhance it, reiterate the key strengths of the NDI-Hi, such as its high reliability and validity. Highlight the broader impact of having a validated Hindi version of the NDI, such as its potential to inform public health policies and improve clinical practices in rural India. Additionally, suggest specific applications of the NDI-Hi in both clinical and research settings, emphasizing its role in enhancing the understanding and management of neck pain among Hindi-speaking populations.

*Authors’ response*

 We have amended changes accordingly.

 Regards,

 Thank you

# Reviewer report: Reviewer response for version 1

**DOI:** 10.5256/f1000research.156005.r291968

**Published:** 2024-07-05

**Authors:** Prem Venkatesan

**Affiliations:** 1 Manipal College of Health Professions, Bangalore, India

Dear Editor,

 Thank you for providing me the opportunity to review the manuscript titled: “Adaption and psychometric evaluation of the Hindi version of Neck Disability Index in the rural population of Northern India: A cross cultural study”

 I have a few concerns about this manuscript:

**Abstract**

1. The background of the study is not convincing enough, this can be written in a more acceptable way
2. The different sample sizes used in the manuscript are absent in the methods section.

**Introduction**

1. The introduction contains a good number of facts and findings, but it looks jumbled up and it doesn’t properly lead to the need for the study. This can be improved by properly articulating the facts.
2. Reference Princpal et al.m is wrongly cited.

**Methods**

1. This study is mentioned as a cross-cultural study that has studied the adoption and psychometric evaluation of the NDI scale, But it is mentioned as an observational study. This has to be cross-checked as cross-cultural studies are not purely observational studies and adoption and psychometric evaluations are mostly methodological studies. This has to be clarified.
2. How was the sample size estimated?
3. Figure 1: Incomplete
4. The data was collected from the subjects after 48 hours, and some justification was given in the discussion. Please provide the evidence for this. Has any evidence suggested that collecting data in 48 hours can give a better outcome? If yes please do justify it in the methodology section also.
5. The sample size to be used for psychometric evaluation and test-retest reliability has to be similar. However, there is a disparity between the numbers used. What is the justification for the change in sample size?
6. It was mentioned that the gender-specific analysis was done. I couldn’t find any analysis.
7. What was the purpose of the pilot study?

**Results**

1. Please align the tables corresponding to the findings. The current presentation makes it tough to correlate the findings.

**Discussion**

1. The discussion can be made better by stressing the findings. This study was done on the shortcomings of the previous study. When the primary aim of this study was to address the shortcomings, the results can also be specific to those.
2. This study has only reported internal consistency and validity, but the aim of the study was not at all discussed with facts and findings.

**References**

1. Two studies with the author name “Study” are wrongly cited.

**General Comments**

1. The manuscript needs language editing and grammatical correction.
2. There is a lack of coherence and cohesion throughout the text.

Is the work clearly and accurately presented and does it cite the current literature?

Partly

If applicable, is the statistical analysis and its interpretation appropriate?

Yes

Are all the source data underlying the results available to ensure full reproducibility?

Yes

Is the study design appropriate and is the work technically sound?

Partly

Are the conclusions drawn adequately supported by the results?

Yes

Are sufficient details of methods and analysis provided to allow replication by others?

Partly

Reviewer Expertise:

My area of expertise is musculoskeletal pain and cardiopulmonary rehabilitation

I confirm that I have read this submission and believe that I have an appropriate level of expertise to confirm that it is of an acceptable scientific standard, however I have significant reservations, as outlined above.

# Response

**Published:** 2024-07-17

**Authors:** Dr Mohammad Sidiq

Dear Editor,

 We express our sincere appreciation to the editor for the time and interest in this study.

 Dear reviewers,

**We appreciate and thank you for the valuable comments, suggestions, and interest in this research paper.**

 We have amended the corrections according to the comments and suggestions provided. In addition, Spelling, grammar, and sentence structure errors have been corrected and the manuscript is revised based on the journal style requirements.

*All the authors agreed on the changes made in response to the reviewer's comments.*

*Please find our responses below:*

 With regards,

 Dr Mohammad Sidiq

***Reviewer 1 comment***

**Abstract**

1. The background of the study is not convincing enough, this can be written in a more acceptable way.
2. The different sample sizes used in the manuscript are absent in the methods section.

*Authors’ response*

 Thank you, the changes are amended in the background section accordingly.

 We recruited n = 30 individuals for cognitive debriefing & n = 211 for the psychometric testing. Both samples are now mentioned in methods section of abstract (lines 43 - 44) and manuscript (methods-participant sub-section – lines 146-147). (Methods, Inclusion and exclusion criteria-Subsection; lines 156 -164).

***Reviewer 1 comment***

**Introduction**

1. The introduction contains a good number of facts   and findings, but it looks jumbled up and it doesn’t properly lead to the need for the study. This can be improved by properly articulating the facts.
2. Reference Princpal et al.m is wrongly cited.

 
*Authors’ response*

 The changes are now amended in the introduction section to improve the cohesion and lead towards the need for the study. (Introduction section, page 4 lines 89 – 95, lines 120 -126)

 The reference is now modified accordingly.

***Reviewer 1 comment***

**Methods**

1. This study is mentioned as a cross-cultural study that has studied the adoption and psychometric evaluation of the NDI scale, But it is mentioned as an observational study. This has to be cross-checked as cross-cultural studies are not purely observational studies and adoption and psychometric evaluations are mostly methodological studies. This has to be clarified.
2. How was the sample size estimated?
3. Figure 1: Incomplete
4. The data was collected from the subjects after 48 hours, and some justification was given in the discussion. Please provide the evidence for this. Has any evidence suggested that collecting data in 48 hours can give a better outcome? If yes please do justify it in the methodology section also.
5. The sample size to be used for psychometric evaluation and test-retest reliability has to be similar. However, there is a disparity between the numbers used. What is the justification for the change in  sample size?
6. It was mentioned that the gender-specific analysis was done. I couldn’t find any analysis.
7. What was the purpose of the pilot study?

*Authors’ response*

1.
  1. Thank you, as per the suggestion we have removed the observational study design. And amended changes related to the methods used (methods, lines 135-136).
  2. Considering the lack of specific guidelines and different authors used different formula to estimate sample size like; factor analysis formula, Kaiser-Meyer-Olkin sampling adequacy (values ranging from 0.50 to 0.60), regression formula and item-sample ratio estimation We intended to include all eligible patients with CNSNP attending both the study center’s during the study period to improve the statistical estimations and consistency of the results. (Methods section, lines 165 -171). A total of 220 respondent consented and 9 participants either did not complete the questionnaire or the data sheet was not complete.
  3. The figure 1 is changed now accordingly
  4. Thank you for the suggestion, we have amended the changes accordingly (Methods section; lines 262 -265)

“test-retest re-administration was set at 48 hours, which is a minimum duration that is likely to result in minimal alterations in the patient's clinical condit110ion and minimal memory-related effects(Marx et al., 2003).Yes, considering the difficulty and constraints (response rate) in inviting the respondents to attend face to face interview prospectively for the 2 ^nd^ time, we used the recommended “minimum sample size required for two-tailed Cronbach’s alpha test was calculated using the Bonet formula based on assumptions” . Hence, test-retest was conducted using n = 110. The same was prospectively informed and approved by the MAPI Research Trust.

1.
  1. Thank you for pointing out, we have removed the statement now.
  2. The purpose of Pilot study and cognitive debriefing is now mentioned in the method section (lines 235 -242).

***Reviewer 1 comment***

**Results**

1. Please align the tables corresponding to the findings. The current presentation makes it tough to correlate the findings.

*Authors’ response*

 Changes done accordingly, thank you

***Reviewer 1 comment***

**Discussion**

1. The discussion can be made better by stressing the findings. This study was done on the shortcomings of the previous study. When the primary aim of this study was to address the shortcomings, the results can also be specific to those.
2. This study has only reported internal consistency and validity, but the aim of the study was not at all discussed with facts and findings.

*Authors’ response*

 Yes, thank you for reminding us of our primary purpose of addressing the methodological gap of previous work.

 We have now amended the changes and added content according to your valid suggestions. (Discussion section, lines 353 – 364)

*“* *it is important to the commonly used NDI raw score is not linear and should likely not be used in interpretation. Hence, it is recommended to transform the raw NDI score into a linear measure (convert to 100) prior to data analysis. The lack of methodologies related to score transformations, hypothesis tested, dimensionality, and testing of validity in the previous work did not permit a meaningful comparison with findings of this study”.*

 Reference; Hung M, Cheng C, Hon SD, Franklin JD, Lawrence BD, et al. Challenging the norm: further psychometric investigation of the Neck Disability Index. Spine J. 2015. doi:10.1016/j.spinee.2014.03.027.

 And, the same is mentioned as well. Please suggest us if the content added is justifying. Thank you again for pointing out.

***Reviewer 1 comment***

**References**

1. Two studies with the author name “Study” are wrongly cited.

*Authors’ response*

 Done accordingly
